# Integrating zooprophylaxis, insecticide-treated livestock and mosquito nets for malaria control: a One Health proposal

**DOI:** 10.1093/trstmh/trag036

**Published:** 2026-04-24

**Authors:** Gbeminiyi R Otolorin, Oyelola A Adegboye, Charibu H Dishon, Hassana I Dunka, Adanu W Adanu, Cecilia O Ihieri, Emma S McBryde

**Affiliations:** College of Medicine and Dentistry, James Cook University, Townsville, QLD 4811, Australia; Australian Institute of Tropical Health and Medicine, James Cook University, Townsville, QLD 4811, Australia; Centre for Tropical Biosecurity, James Cook University, Townsville, QLD 4811, Australia; Department of Veterinary Public Health and Preventive Medicine, Faculty of Veterinary Medicine, University of Jos, Jos, Plateau State 930001, Nigeria; College of Medicine and Dentistry, James Cook University, Townsville, QLD 4811, Australia; Centre for Tropical Biosecurity, James Cook University, Townsville, QLD 4811, Australia; Menzies School of Health Research, Charles Darwin University, Darwin, NT 0811, Australia; Department of Veterinary Public Health and Preventive Medicine, Faculty of Veterinary Medicine, University of Jos, Jos, Plateau State 930001, Nigeria; Department of Veterinary Public Health and Preventive Medicine, Faculty of Veterinary Medicine, University of Jos, Jos, Plateau State 930001, Nigeria; Department of Veterinary Public Health and Preventive Medicine, Faculty of Veterinary Medicine, University of Jos, Jos, Plateau State 930001, Nigeria; Department of Family Medicine, Federal Teaching Hospital, Owerri, Owerri, Imo State 460281, Nigeria; College of Medicine and Dentistry, James Cook University, Townsville, QLD 4811, Australia; Australian Institute of Tropical Health and Medicine, James Cook University, Townsville, QLD 4811, Australia; Centre for Tropical Biosecurity, James Cook University, Townsville, QLD 4811, Australia

**Keywords:** insecticide-treated livestock, insecticide-treated net, integrated vector management, malaria, One Health, zooprophylaxis

## Abstract

Malaria transmission persists despite widespread use of insecticide-treated nets (ITNs) due to multiple factors, including outdoor and early-evening biting and insecticide resistance. In this context, zooprophylaxis, which strategically uses livestock to divert mosquitoes from humans, has shown mixed epidemiological effects. Conversely, treating livestock with topical or systemic insecticides (known as insecticide-treated livestock [ITL]) has been demonstrated to significantly reduce mosquito survival. Combining ITL and zooprophylaxis with ITNs could improve vector control for species that feed outdoors and prefer animal hosts. This short communication reviews an integrated approach within integrated vector management and proposes a practical malaria One-Health control strategy.

## Introduction

**Figure 1:**
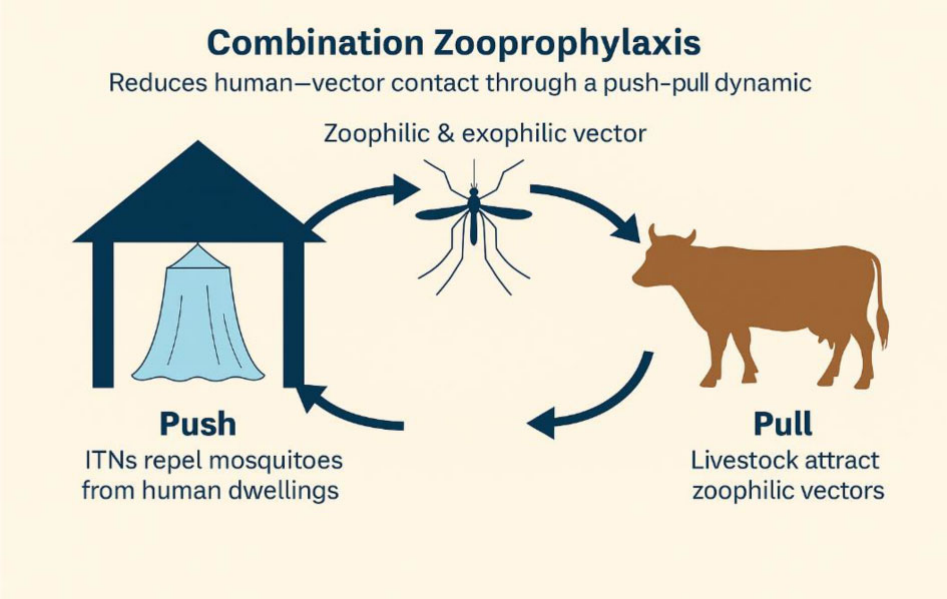
Integrated malaria-control strategies illustrating the roles of ITNs and zooprophylaxis.

Malaria remains one of the most significant vector-borne diseases worldwide, and the progress achieved over the past 20 y is now threatened by rising insecticide resistance and the adaptable behaviour of *Anopheles* mosquitoes.^1^ The main strategies currently relied upon, such as insecticide-treated nets (ITNs) and indoor residual spraying (IRS), are highly effective against mosquitoes that bite indoors at night. However, their effectiveness is increasingly limited by behavioural changes in mosquito populations, including early evening and outdoor biting, which enable continued transmission despite widespread use of ITNs and IRS.^2^ Persistent malaria transmission is often driven by vector species that preferentially feed outdoors or on animals. While interventions such as ITNs remain highly effective against endophagic and endophilic species like *Anopheles gambiae* s.s. and *Anopheles funestus* s.s., they show limited impact on more opportunistic vectors such as *Anopheles arabiensis*, which frequently bite non-human hosts and exhibit exophagic behaviour.^3^ This ecological flexibility helps these mosquitoes thrive even when ITN coverage is high. Combination zooprophylaxis integrates livestock management with conventional vector-control tools, such as ITNs, to create a push-pull dynamic that reduces human–vector contact. In this system, ITNs may exert a repellent effect on predominantly zoophilic and exophilic mosquito species, reducing entry into human sleeping spaces, while livestock housed separately attract host-seeking vectors such as *Anopheles arabiensis*, thereby creating a push-pull dynamic (Fig. 1). For this strategy to be effective, three essential conditions must be met: (i) the presence of a zoophilic and exophilic vector; (ii) spatial separation between animal shelters and human dwellings; and (iii) the integration of livestock treatment or ITN/IRS co-interventions to enhance vector mortality.^4,5^ Considering ecological and operational factors, this paper proposes an integrated framework combining zooprophylaxis, insecticide-treated livestock (ITL) and ITNs as a complementary malaria-control strategy. Instead of presenting new empirical trial data, this manuscript offers a conceptual and future-oriented discussion on how these interventions could be strategically aligned within endemic settings. The main goal is to examine the potential epidemiological impact of integrating host diversion with increased vector mortality mechanisms. Specifically, this paper aims to (i) compile emerging evidence on zooprophylaxis and ITL within a vector ecology context; (ii) explore how these approaches may complement existing ITN-based control strategies; and (iii) foster critical discussion and future research directions on the role of livestock-focused interventions in malaria-elimination efforts.

## Methods

A focused literature review was carried out using PubMed, Web of Science and Scopus, supplemented by targeted Google Scholar searches. Search strings combined terms with Boolean operators, such as (‘insecticide-treated nets’ OR ITNs) AND (‘insecticide-treated livestock’ OR ITL OR zooprophylaxis) AND (‘malaria’ OR ‘malaria vector control’) AND (Anopheles OR ‘host diversion’ OR livestock). Studies were eligible if they examined livestock-based malaria-control strategies, vector–host interactions, entomological or epidemiological outcomes, or integration with established interventions. Titles and abstracts were screened for relevance, followed by full-text review where necessary. Extracted information was synthesized thematically to identify integration frameworks and research gaps relevant to the evaluation of ITL within malaria-control strategies.

## Evidence-based research

### Zooprophylaxis

Zooprophylaxis in malaria control involves using domestic animals to attract mosquitoes away from humans and has been proposed as a natural method to help control malaria.^4^ In regions where mosquitoes such as *Anopheles arabiensis* thrive, having cattle or goats nearby can significantly reduce the number of bites humans receive. The effectiveness of zooprophylaxis is context-dependent. It tends to be most beneficial in areas where the dominant mosquito species are highly zoophilic and livestock are kept at a reasonable distance from human dwellings during peak biting times. Complementary protective measures, such as ITNs, can enhance this effect but may also mask its independent contribution. However, in ecosystems where several mosquito species coexist, each with different host preferences and behaviours, the outcomes become unpredictable. Under such complex ecological conditions, close proximity of livestock to humans can increase vector density and human exposure, a phenomenon known as zoopotentiation. These findings emphasize the need for context-specific evaluation of zooprophylaxis, considering local vector composition, host abundance and spatial arrangement, before it is adopted as a large-scale control strategy.^6^

### Livestock-based malaria control using insecticides and systemic endectocides

ITL extends zooprophylaxis by adding a lethal component: mosquitoes feeding on treated animals die or exhibit reduced fecundity. This can be achieved through topical application of pyrethroids or fipronil, or via systemic endectocides such as ivermectin or eprinomectin.^7,8^ Research and semi-field trials have shown that treated cattle can significantly decrease the survival and reproduction rates of *Anopheles* mosquitoes, with effects lasting several days post-treatment.^7^ Although considerable progress is still needed to turn ITL from an experimental concept into a practical malaria-control method, its potential benefits deserve consideration even at this early stage. Implementing ITL would require close collaboration between human and animal health sectors, fostering valuable opportunities for resource sharing and coordinated efforts. Such cross-sector cooperation embodies the One Health principle, encouraging joint efforts towards improved health outcomes for both humans and animals.^9^ While the entomological benefits are clear, evidence from epidemiological studies remains somewhat tentative. Practical considerations, such as product costs, residue management and waiting periods for meat and milk, require further investigation before wider implementation can be realized. Nevertheless, ITL remains a promising supplementary strategy, particularly in areas where species that bite animals are prevalent and outdoor transmission continues, even with high coverage of ITNs.

### Interaction with ITNs

ITNs remain the mainstay of malaria prevention, mainly targeting those troublesome indoor, nocturnal-biting mosquitoes. However, prolonged use of ITNs exerts significant pressure on these vector populations, resulting in behavioural changes such as feeding outdoors or on animals.^10^ This shift has been observed in species such as *An. arabiensis, Anopheles albimanus* and *Anopheles farauti*.^10^ Such adaptability paves the way for combined strategies that utilize both ITNs and ITL to address both indoor and outdoor transmission simultaneously.^11^

Mathematical and agent-based models suggest that combining ITNs with livestock-based interventions (such as ITL) may further reduce malaria transmission, especially in settings with high cattle-to-human ratios and zoophilic vectors, although precise quantitative estimates vary and remain subject to modelling assumptions.^12^ The idea is two-fold: (i) ITNs can steer host-seeking mosquitoes towards livestock; and (ii) these mosquitoes can then be wiped out through contact with either insecticide-treated or medicated animals.^9^

Although theoretical and modelling studies suggest that combining ITL with ITNs could enhance malaria control, real-world evidence remains limited. To date, large-scale, population-level field trials evaluating the combined epidemiological impact of ITNs and ITL are lacking. Most evidence for livestock-based interventions comes from modelling, small-scale trials or entomological endpoints, rather than clinical malaria-incidence data.^9,13^ This ongoing evidence gap highlights the need for well-designed community-level evaluations of integrated interventions, including ITL, within the frameworks of One Health and integrated vector management (IVM). The absence of such studies reveals a significant research gap and provides an opportunity to assess how livestock-based interventions can complement ITN and IRS coverage in real-world settings.

### Implementation challenges and context specificity of ITL within integrated vector-control frameworks

Field evaluations of ivermectin-treated cattle demonstrate that, at currently approved dosing regimens, mosquitocidal activity is substantial but transient, with effectiveness declining over time and therefore insufficient to sustain long-term interruption of malaria transmission without repeated administration or the use of long-acting formulations.^14^

In malaria-transmission systems, heterogeneity in mosquito-feeding behaviour may further limit the effectiveness of ITL. Vectors with stronger zoophilic tendencies are more likely to be exposed to insecticides applied to animals, whereas more anthropophagic subpopulations may avoid exposure and continue contributing to human–malaria transmission.^13^ This behavioural differentiation could result in uneven suppression of vector populations and constrain the overall impact of ITL, indicating the need for further research to better understand how feeding-behaviour heterogeneity influences malaria-control outcomes.^15^ Additionally, a potential limitation of ITL is that commonly used insecticides are behaviourally neutral or repellent to mosquitoes and therefore may not increase mosquito attraction to animals, limiting mosquito contact and reducing the overall impact of the intervention unless combined with additional attractant strategies.^13,16^

The adoption of ITL is also limited by various practical and structural barriers. Restricted access to veterinary services, supply chains for approved insecticides or endectocides, and trained personnel, can further hinder implementation, especially in rural or resource-limited areas.^17^ Regulatory restrictions related to veterinary drug approval, including residue regulations and required withdrawal periods for meat and milk, can significantly restrict the range of allowed compounds and application schedules.^9^ Additionally, variation in insecticide efficacy can stem from formulation-specific factors, such as delivery method and chemical composition, as well as host-specific traits, including animal species, age and metabolic capacity. Furthermore, farmer acceptance may be impeded by concerns about animal health, productivity and marketability.^18^

The effectiveness of malaria vector-control interventions is highly context-specific and varies substantially by geography, vector ecology and human behaviour, with ITNs, ITL and zooprophylaxis each suited to distinct transmission settings across sub-Saharan Africa and Asia. ITNs provide maximal protection in regions dominated by highly endophilic and endophagic vectors such as *Anopheles gambiae* s.s. in West and Central Africa, where most malaria transmission occurs indoors during sleeping hours.^19,20^ However, their effectiveness is reduced in parts of Southeast Asia and the Sahel where vectors such as *Anopheles maculatus, Anopheles subpictus* and *An. arabiensis* exhibit substantial outdoor or early-evening biting, leading to significant residual transmission, despite high net coverage.^21,22^

ITL is most likely to be effective in settings where malaria vectors exhibit zoophilic or opportunistic feeding behaviour and where livestock are plentiful and deeply integrated into daily human life, such as pastoral and agro-pastoral regions of East Africa, the Sahel and parts of The Gambia, where *An. arabiensis* is a dominant vector and outdoor transmission continues beyond the protective scope of ITNs.^13,23^

Although zooprophylaxis has yielded highly variable outcomes across regions, it reduces malaria risk in some ecological contexts but can increase transmission elsewhere through zoopotentiation when livestock are kept close to human sleeping areas. Classic studies from Pakistan and Ethiopia demonstrate that cattle ownership or co-housing with livestock can raise the malaria risk by increasing vector density or human biting rates, highlighting the importance of spatial separation between humans and animals.^24,25^

Together, these patterns suggest that effective malaria control requires geographic and ecological stratification: prioritizing ITNs in stable indoor-transmission areas; deploying ITL in regions characterized by zoophilic vectors and pastoral lifestyles; and carefully managing livestock placement to prevent zoopotentiation.

### Evidence from vector-control strategies supporting ITL within integrated vector control

Although direct empirical evidence for ITL as a malaria intervention remains limited, the underlying principle aligns with established vector-control strategies where interventions target non-human hosts or environments to decrease vector populations and protect humans from vector-borne diseases.^26^ Well-known examples include the use of insecticide-treated cattle and odour-baited targets to suppress tsetse fly populations and reduce human African *trypanosomiasis*, despite cattle or artificial baits being the treated entities and humans the primary beneficiaries.^27,28^ Similarly, insecticide-treated dog collars have been employed to control sand flies and lower human visceral leishmaniasis, even although dogs are the treated species and humans are the protected population.^29^

ITL works at the community level by targeting mosquitoes during blood feeding on livestock, effectively turning animals into lethal decoys that decrease vector survival and transmission potential rather than relying solely on passive diversion like in traditional zooprophylaxis, which may produce uncertain epidemiological outcomes.^24^ Larval source management offers a similar approach, where aquatic habitats are treated to reduce mosquito populations and protect humans without direct human exposure to the intervention.^30^ Collectively, these examples show that indirect, non-human–targeted interventions are a well-established part of IVM and support the biological plausibility of ITL as a complementary strategy alongside ITNs, rather than as a substitute for them.

Importantly, ITNs may further improve the effectiveness of non-human–directed interventions such as ITL by reducing human availability to host-seeking mosquitoes, thereby increasing the likelihood that vectors seek alternative blood meals from treated livestock.^19^ By decreasing successful mosquito–human contact, ITNs directly reduce malaria transmission while also enhancing the effect of ITL by redirecting mosquito feeding towards animals that act as lethal decoys, thereby increasing vector mortality rather than simply switching hosts.

### Potential risks and interaction effects of ITNs, ITL and zooprophylaxis

The implementation of ITNs, ITL and zooprophylaxis requires careful consideration of potential risks and interaction effects, which may be additive, synergistic or antagonistic depending on the ecological and operational context. Synergistic effects are most likely when ITNs are combined with ITL in settings characterized by zoophagic or opportunistic *Anopheles* populations and residual outdoor transmission, as ITNs reduce human availability while ITL increases mosquito mortality during livestock feeding, thereby converting diversion into lethal contact.^9,19^

By contrast, antagonistic effects may occur when zooprophylaxis is used without insecticidal treatment of livestock or when animals are kept close to human sleeping areas, potentially increasing vector density and malaria risk through zoopotentiation.^6,24^ Other risks include incomplete livestock treatment coverage, the development of insecticide resistance and environmental impacts of veterinary parasiticides, emphasizing the importance of resistance monitoring, environmental assessment and deploying measures tailored to specific contexts.^26^ Overall, the existing literature supports using ITNs as the primary intervention, with ITL and managed zooprophylaxis applied selectively as supplementary tools where vector ecology, livestock density and capacity for implementation are appropriate.

### Research-informed operational recommendations for combined zooprophylaxis in high livestock-density settings

Based on accumulated evidence from experimental, field and modelling studies, the implementation of combined zooprophylaxis approaches—integrating ITL with ITNs—in areas of high livestock density should be accompanied by a structured monitoring framework that reflects both mosquito diversion dynamics and vector mortality outcomes.^9,31^ Existing research suggests that monitoring frameworks should prioritize indicators capable of distinguishing beneficial diversion from harmful zoopotentiation, while also capturing reductions in vector survival and transmission potential. It is recommended that environmental risk assessments of veterinary parasiticides proposed for ITL are conducted through region-specific, population-level field studies in malaria-endemic settings. Such evaluations should consider local climatic conditions, vegetation, dung fauna and soil-water dynamics, as evidence from farm-level studies in temperate regions and individual-animal use may not accurately reflect environmental impacts under large-scale ITL deployment.^9^

In the context of combined strategies such as ITL, ITNs and zooprophylaxis, periodic measurement of the Human Blood Index (HBI) can help to determine whether host-seeking mosquitoes are being diverted away from humans towards treated livestock or alternative hosts, making it a practical entomological indicator for evaluating intervention effectiveness.^32^ Identifying the blood-meal origins of female *Anopheles* mosquitoes is crucial for informing the development and optimization of vector-control interventions, with zoophagic indices (such as bovine or ovine blood indices) providing complementary insights into livestock-directed feeding patterns.^33^

Although this study does not generate new empirical data or undertake formal quantitative analyses, it integrates and critically appraises existing experimental and field evidence to clarify the biological and operational contexts in which zooprophylaxis and ITL are most plausibly effective. In particular, it highlights that impact depends on vector host preference (zoophilic or opportunistic *Anopheles* spp.), as well as implementation factors such as livestock availability, treatment coverage and the spatial arrangement of livestock relative to human dwellings. Rather than providing definitive effect estimates, this article offers a conceptual framework to support hypothesis generation and guide future empirical research. Evidence from blood-meal studies shows that reductions in the HBI, alongside increases in zoophagic indices, provide measurable indicators of beneficial diversion. However, inappropriate livestock placement may lead to zoopotentiation and increased malaria risk.^6,34^ By combining findings from ITNs, ITL and traditional zooprophylaxis, the short communication emphasizes where livestock-based interventions are biologically feasible and programme-justified, rather than suggesting they are universally effective.

## Conclusion

This Short communication highlights the promising potential of combining zooprophylaxis, ITL and ITNs within a unified One Health approach to combat malaria. Evidence from early studies indicates that livestock can affect mosquito behaviour, and treating these animals with insecticides or endectocides can notably reduce the survival, reproduction and sporozoite development of *Anopheles* mosquitoes. However, despite these biologically plausible findings, there are still no definitive randomized or long-term studies demonstrating the epidemiological impact of ITL or its combined effects with ITNs. This absence of evidence creates a vital gap in the global vector-control strategy, particularly as outdoor and zoophilic transmission continues to sustain residual malaria in areas with high ITNs and IRS coverage.

The livestock-based approach aligns with the WHO’s IVM principles and exemplifies the One Health concept by utilizing veterinary resources for public health benefits. It redefines livestock from passive entities to active participants in sustainable malaria-control systems. Future research should include cluster-randomized or stepped-wedge trials comparing strategies: ITN-only, ITL-only, a combination of ITN and ITL, and control groups. For ITL to become a feasible and mainstream malaria-prevention strategy, several essential research priorities must be systematically addressed first. Initially, rigorous community-level evaluations are needed to compare integrated livestock-based approaches with standard vector-control strategies. Future trials should explicitly include zooprophylaxis as a designated comparator arm, enabling structured assessments of additive, synergistic or potentially antagonistic effects when combined with ITNs or other established interventions. Additionally, comprehensive entomological surveillance frameworks must accompany such evaluations. These should involve long-term monitoring of vector density, parity rates, sporozoite prevalence and insecticide-susceptibility patterns. Molecular blood-meal analysis is crucial for measuring host diversion dynamics, while behavioural and genomic studies are necessary to identify species-specific adaptive responses to livestock-targeted insecticides. Furthermore, spatial and ecological threshold analyses are essential to identify conditions under which zooprophylaxis offers protection rather than risk. Practically, such analyses could be carried out through structured experimental or quasi-experimental designs that vary livestock–household proximity in controlled and measurable ways. For example, cluster-based field trials might assign villages or subvillage units to specified livestock corralling distances (e.g. <10 m, 10–30 m, >30 m from human sleeping areas), while maintaining consistent livestock density. Alternatively, phased relocation protocols could gradually move corrals across transmission seasons, allowing comparisons of clusters based on vector density, human-biting rate and malaria incidence before and after adjustments for distances. Furthermore, epidemiological modelling and transmission-dynamics research should quantify the long-term population-level impact of ITL under various transmission intensities, vector behaviours and intervention coverage levels. Without such modelling, the scalability and sustainability of ITL remain uncertain. Only after thoroughly addressing these ecological, entomological, spatial and epidemiological uncertainties can ITL be responsibly evaluated as a candidate for wider policy adoption. Currently, ITL should be viewed as an emerging and promising but still investigational strategy within the evolving landscape of integrated and One Health-oriented malaria control. Finally, transmission-dynamics modelling is needed to quantify the long-term population-level impact and scalability of ITL under varying transmission intensities, vector host preference indices and intervention coverage levels. Mechanistic models that incorporate feeding plasticity, insecticide decay kinetics, livestock turnover and vector behavioural adaptation are particularly important to assess sustainability. Until these ecological, spatial, entomological and epidemiological uncertainties are resolved, ITL should be regarded not as a mainstream replacement for existing strategies, but as a context-specific, emerging adjunct whose policy relevance remains contingent upon rigorous evidence generation.

## Data Availability

No new data were generated during this study.
